# Genome-wide identification and expression analysis of the *DA1*-*like* gene family in melon (*Cucumis melo*)

**DOI:** 10.3389/fpls.2025.1742747

**Published:** 2026-01-20

**Authors:** Mingzhen Wang, Rui Wang, Hualin Zhang, Qianxing Wu, Mingming Dong, Yunhai Li, Lian Wu, Xiaofeng Yang

**Affiliations:** 1 School of Breeding and Multiplication (Sanya Institute of Breeding and Multiplication), School of Tropical Agriculture and Forestry, Hainan University, Sanya, China; 2 State Key Laboratory of Plant Cell and Chromosome Engineering, Chinese Academy of Sciences (CAS) Centre for Excellence in Molecular Plant Biology, Institute of Genetics and Developmental Biology, Chinese Academy of Sciences, Beijing, China

**Keywords:** *DA1-like* gene family, gene expression, *melo*, organ development, phylogenetic analysis

## Abstract

The *DA1* (DA means “large” in Chinese) *-like* gene family represents a class of key regulators that control organ and seed size in plants, yet its characteristics and functions in melon (*Cucumis melo*) remain unexplored. In this study, we performed a genome-wide identification and characterization of the *CmDA1* gene family in melon. A total of four *CmDA1* genes were identified and found to be unevenly distributed across chromosomes 3, 8, 10, and 11. Phylogenetic analysis grouped the *CmDA1* genes into three distinct subgroups with their orthologs from *Arabidopsis thaliana* (*Arabidopsis*) and *Oryza sativa* (rice). Structural analysis revealed high conservation within subfamilies, particularly in the characteristic UIM and LIM domains. The presence of hormone-responsive *cis*-elements, combined with divergent gene structures, suggests complex transcriptional regulation of the *CmDA1* genes. Expression profiling based on RNA-seq data and qRT-PCR validation demonstrated that *CmDA1* genes exhibit tissue-specific and fruit development-stage-specific expression patterns. Notably, *CmDA1–3* showed broad and high expression across multiple vegetative tissues and maintained consistently high expression throughout fruit development from 2 to 50 days after pollination. These findings provide a foundational resource for the DA1 family in melon and position *CmDA1–3* as a priority candidate for functional validation of its role in fruit size regulation.

## 1 Introduction

Melon (*Cucumis melo*) is an important horticultural crop belonging to the Cucurbitaceae family. As a diploid plant with 12 chromosomes (2n = 2x = 24) (Xu et al., 2022), its global production exceeds 30 million metric tons annually across more than 100 countries, establishing it as a major crop in tropical and subtropical regions (Shahwar et al., 2023). The fruit is highly valued for its economic importance and nutritional quality, being rich in sugars, vitamins, and antioxidants (Mayobre et al., 2021). Among the key agronomic traits, fruit size and shape are particularly crucial as they directly influence yield and market acceptance (Kesh and Kaushik, 2021). These traits are governed by multiple genes and involve complex cellular processes, including cell division and expansion. Therefore, elucidating the genetic mechanisms controlling these traits is essential for molecular breeding aimed at improving melon varieties.

The *DA1* gene family plays a central role in regulating plant organ size by encoding ubiquitin receptors that act as negative regulators of seed and organ development (Keren et al., 2020; Vanhaeren et al., 2020; Gong et al., 2022; Zhou et al., 2024). In *Arabidopsis*, the gain-of-function mutant *da1-1*, which carries an R358K substitution, produces larger seeds and organs due to an extended period of cell proliferation (Li et al., 2008; Li and Li, 2014). Structurally, DA1 proteins typically contain N-terminal ubiquitin-interacting motifs (UIMs) and a C-terminal LIM domain, functioning through the ubiquitin–proteasome pathway (Dong et al., 2017). *DA1* directly interacts with DA2, an E3 ubiquitin ligase homologous to rice GW2, to promote the degradation of target substrates and thus repress cell division (Xia et al., 2013; Dong et al., 2017). Furthermore, *DA1* fine-tunes organ growth by interacting with the deubiquitinating enzyme UBP15/SOD2, which modulates its protein stability (Du et al., 2014). Functional redundancy exists between *DA1* and its paralogs, the *DA1*-*RELATED* (*DAR*) genes; while single T-DNA insertion mutants exhibit subtle phenotypes, the *da1*/*dar1* double mutant shows pronounced organ enlargement, phenocopying the *da1–1* mutant (Li et al., 2008). Beyond growth regulation, *DA1*-*like* genes are implicated in stress responses and leaf senescence, underscoring their pleiotropic functions (Vanhaeren et al., 2017).

Extensive research on *DA1* and its related genes (*DARs*) across both dicot and monocot crops confirms their conserved role as growth regulators, while also revealing increasing complexity in their regulatory mechanisms. In rapeseed (*Brassica napus*), reducing *BnDA1* activity increases seed size and plant biomass, confirming its function as a growth suppressor with breeding value (Wang et al., 2017). Research in maize reveals that gene dosage and specific mutations lead to distinct phenotypic outcomes. High expression levels of wild-type *ZmDA1* or *ZmDAR1* reduce seed size, whereas the expression of mutant versions carrying the R358K substitution (analogous to the *Arabidopsis da1–1* allele) results in larger kernels and higher yield (Xie et al., 2018). Interestingly, when scientists created a triple mutant lacking three DA1-type genes using CRISPR, the plants showed only minor size changes, indicating that other genes may compensate in maize (Gong et al., 2022). Research in wheat supports the breeding potential of this pathway; modulating *TaDA1* activity influences kernel size, and a specific natural haplotype (*TaDA1*-A-HapI) is associated with consistently larger grains, highlighting its utility in breeding programs (Liu et al., 2020). Similarly, in rice, a single amino acid substitution (R310K) in *OsDA1* enhances grain size and yield potential (Li et al., 2024). Although *DA1* genes control organ size across diverse species and their orthologs have been identified in several crops, a systematic genome-wide characterization of this gene family has not been conducted in melon.

In this study, we performed a systematic genome-wide analysis of the *DA1*-*like* gene family in melon. We comprehensively characterized the *CmDA1* gene members, including their phylogenetic relationships, gene structures, chromosomal distributions, and promoter cis-elements. Furthermore, we analyzed their expression profiles across different tissues and fruit developmental stages, with key patterns validated by quantitative real-time PCR (qRT-PCR) in “the melon inbred line HN15”. This research provides insights into the *DA1* gene family in melon and lays a theoretical basis for the future functional study of individual *CmDA1* members.

## 2 Materials and methods

### 2.1 Identification and physicochemical characterization of the melon *DA1*-*like* gene family

The melon (*Cucumis melo*) DHL92 v4.0 genome sequence, protein sequences, and annotation file were downloaded from the CuGenDB database (http://cucurbitgenomics.org/, accessed on 8 July 2025). The amino acid sequences of *Arabidopsis* DA1 and DA1-related (DAR) proteins were obtained from TAIR (https://www.arabidopsis.org/, accessed on 8 July 2025). We used two methods to identify CmDA1 proteins. First, a Hidden Markov Model (HMM) search was performed using the DA1 domain profile (PF12315) from Pfam (https://www.ebi.ac.uk/interpro/entry/pfam/, accessed on 8 July 2025) (Potter et al., 2018). The melon proteome was searched with HMMER, retaining sequences with E-values ≤ 1e-5 (Ye et al., 2006). Second, a BLASTP search was conducted using the Arabidopsis DA1 and DAR sequences as queries against the melon proteome, with an E-value cutoff of 1e-5. Candidate sequences from both methods were combined. We removed redundant sequences and alternative transcripts, keeping only the longest transcript for each gene. The resulting sequences were verified for the presence of UIM and LIM domains using SMART (http://smart.embl-heidelberg.de/, accessed on 8 July 2025) and NCBI CDD (https://www.ncbi.nlm.nih.gov/Structure/cdd/wrpsb.cgi, accessed on 8 July 2025). Sequences lacking these domains were discarded, yielding the final *CmDA1* gene family members. Physicochemical properties of CmDA1 proteins, including amino acid number, molecular weight, and theoretical pI, were predicted using the ProtParam tool on ExPASy (https://web.expasy.org/protparam/, accessed on 8 July 2025).

### 2.2 Phylogenetic analysis of the *CmDA1* gene family

Protein sequences of DA1 family members were retrieved from *Arabidopsis thaliana*, Cucumis melo, *Oryza sativa* (Rice Genome Annotation Project, http://rice.uga.edu), Cucumis sativus (v3.0, http://cucurbitgenomics.org), and Citrullus lanatus (Watermelon Genome Database, http://www.watermelondb.cn). Multiple sequence alignment was performed using ClustalW within MEGA software (v11.0.13). A phylogenetic tree was constructed using the Neighbor-Joining (NJ) method with the following parameters: 1000 bootstrap replicates, amino acid p-distance model, and partial deletion for gap treatment (site coverage cutoff of 50%) (Kumar et al., 2016). The resulting tree was visualized and annotated using the iTOL online tool (https://itol.embl.de) (Letunic and Bork, 2021). The identified *CmDA1* genes were systematically named based on their phylogenetic clustering patterns and established nomenclature from related species.

### 2.3 Analysis of chromosomal location, conserved motifs, and gene structure of the *CmDA1* genes

The chromosomal positions of the identified CmDA1 genes were extracted from the melon genome annotation file (GFF3) and visualized using the ‘Gene Location Visualize from GTF/GFF’ function in TBtools (v2.310). Conserved motifs in the CmDA1 protein sequences were identified using the Multiple Expectation Maximization for Motif Elicitation (MEME) online tool (http://meme-suite.org/tools/meme, accessed on 10 July 2025) with the following parameters: maximum number of motifs set to 10, and motif width restricted to 6–100 amino acids (Bailey et al., 2009). Protein domains were predicted using the NCBI Conserved Domain Database (https://www.ncbi.nlm.nih.gov/Structure/cdd/wrpsb.cgi, accessed on 10 July 2025) (Marchler-Bauer et al., 2015). Gene structure features (exon-intron organization) were derived from the comparison of the genomic DNA and coding sequences of the *CmDA1* genes. Finally, the phylogenetic tree, conserved motifs, protein domains, and gene structures were integrated and visualized using the “Gene Structure View (Advanced)” module in TBtools (V2.310).

### 2.4 Analysis of gene duplication and synteny relationships

Intraspecific synteny and gene duplication events (including segmental and tandem duplications) among the *CmDA1* genes were analyzed using the One-Step MCScanX tool in TBtools with default parameters (Wang et al., 2012). The results, including chromosomal distributions, gene density, and syntenic relationships, were integrated and visualized in a Circos plot using the Advanced Circos module within TBtools (v2.310) (Krzywinski et al., 2009).

For interspecific synteny analysis, whole-genome protein sequences, and annotation files of *Cucumis melo*, *Arabidopsis*, and *Oryza sativa* were compared using the One-Step MCScanX module in TBtools. The analysis was performed with an E-value threshold of 1e-3 and the number of BLAST hits set to 10. Syntenic relationships were visualized using the Multiple Synteny Plot module, with *CmDA1* gene family members highlighted to identify orthologous gene pairs among the three species.

### 2.5 Analysis of *cis*-regulatory elements in *CmDA1* genes

The 2,000 bp promoter sequences upstream of the transcription start sites of *CmDA1* genes were extracted using TBtools (v2.310). Cis-regulatory elements in these promoter regions were identified using the PlantCARE online database (http://bioinformatics.psb.ugent.be/webtools/plantcare/html/, accessed on 12 July 2025). The cis-regulatory elements identified by PlantCARE were visualized using the “Simple BioSequence Viewer” module in TBtools (v2.310).

### 2.6 Expression analysis of *CmDA1* genes across tissues and fruit development

To investigate the expression patterns of *CmDA1* genes, we utilized publicly available RNA-seq data from BioProject PRJDB6414 (Yano et al., 2018). Our analysis included two main aspects: tissue-specific expression across eight distinct tissues (roots, stems, upper stems, young leaves, mature leaves, flowers, fruits, and tendrils) and developmental expression during fruit maturation at nine time points from 2 to 50 days after pollination. The raw sequencing data underwent comprehensive processing through an established bioinformatics pipeline. Initially, sequence data were converted to FASTQ format using fastq-dump (v3.0.10), followed by quality assessment and filtering with fastp (v0.24.1). Cleaned reads were then aligned to the melon reference genome using HISAT2 (v2.1.0) with appropriate strand-specific parameters. The resulting alignment files were processed with SAMtools (v1.21) for format conversion, and gene expression levels were calculated as FPKM values using custom R scripts. We constructed separate heatmaps with TBtools (v2.310) to display the expression patterns of *CmDA1* genes.

### 2.7 RNA extraction and quantitative real-time PCR analysis

We used the melon inbred line HN15, which was developed by our research group. The mature fruits of the ‘HN15’ inbred line typically reach a single fruit weight of 1000–1500 g. Plants were cultivated under controlled greenhouse conditions with a 16/8−h light/dark cycle and temperatures maintained at 28°C during the day and 18°C at night. To examine the expression of the four *CmDA1* genes, fruit flesh samples were harvested at four developmental stages: 7, 15, 22, and 30 days after pollination (DAP). Roots, stems, and leaves were sampled at the flowering stage. For each tissue type and fruit stage, three biological replicates were taken. All collected samples were immediately frozen in liquid nitrogen and stored at −80°C until RNA extraction.

For RNA extraction, we employed the Spectrum™ Plant Total RNA Kit (Merck KGaA) following the manufacturer’s protocol. RNA quality and concentration were verified using a NanoDrop 2000 spectrophotometer. We then synthesized first-strand cDNA from 1 µg of total RNA using the SweScript All-in-One First-Strand cDNA Synthesis Kit (TRANS, G3337). Specific primers for CmDA1 were designed with Primer Premier 5 software to generate 150–300 bp amplicons.

The qRT–PCR reactions were conducted in 20 µL volumes containing 10 µL of 2× SYBR Green qPCR Master Mix (no ROX) (TRANS, G3320), 1 µL of cDNA template, and 2 µL each of forward and reverse primers. Amplification was performed on a CFX96 Real-Time PCR Detection System (Bio-Rad, USA) using the following program: 95°C for 30 s, then 40 cycles of 95°C for 15 s and 60°C for 30 s. We used the melon EF1α gene as an internal control and calculated relative expression levels according to the 2^-ΔΔCT^ method (Zhang et al., 2018). The experiment included three biological replicates, each with three technical replicates. Statistical analysis was performed using one-way ANOVA in SPSS (v26.0), and graphical representations were prepared with GraphPad Prism 8. All primer sequences are provided in **Supplementary Table S1**.

## 3 Results

### 3.1 Genome-wide identification and characterization of *DA1*-*like* genes in melon

**Figure 1:**
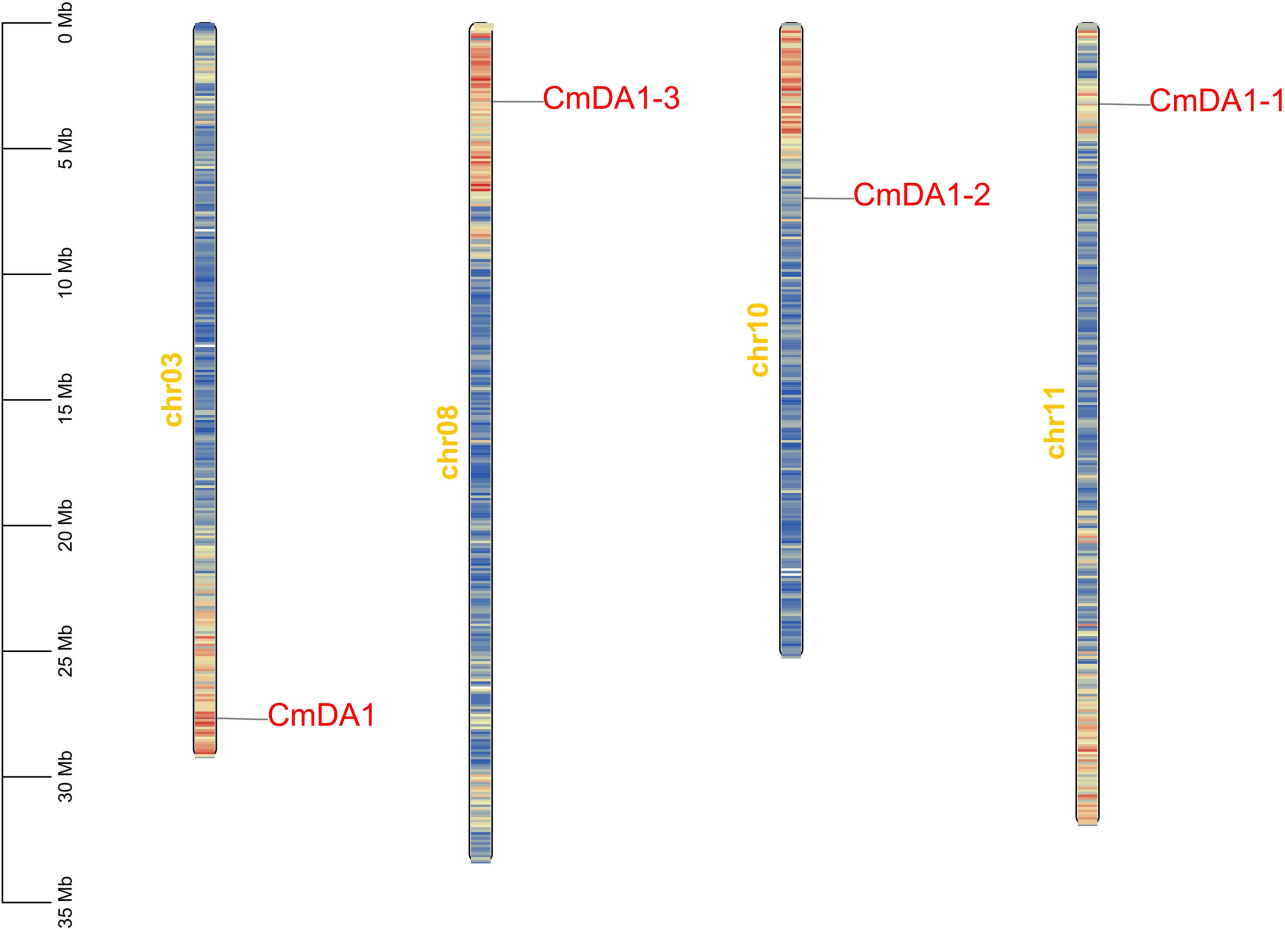
Chromosomal distribution of the 4 identified *CmDA1* genes. Gene positions are mapped to the melon chromosomes (Chr), with the scale bar indicating their physical lengths. Gene density, calculated in 200-kb intervals, is represented by a blue-to-red color gradient, while blank areas denote regions devoid of gene annotations.

We identified four *DA1* genes in the melon genome through a genome-wide analysis. Based on phylogenetic relationships with *Arabidopsis*, these genes were named *CmDA1*, *CmDA1-1*, *CmDA1-2*, and *CmDA1-3*. Analysis of the physicochemical properties revealed variations among the four CmDA1 proteins (**Table 1**). The coding sequence lengths ranged from 1,281 bp to 1,443 bp, encoding proteins of 426 to 480 amino acids. Molecular weights varied from 47.84 kDa to 55.41 kDa, while theoretical isoelectric points spanned from 4.53 to 6.84, indicating differential charge characteristics. Chromosomal mapping showed that the four *CmDA1* genes were distributed across chromosomes 3, 8, 10, and 11, with each gene located on a separate chromosome (**Figure 1**).

**Table 1:** Genome-wide identification and characterization of the *CmDA1* gene family in melon.

Gene name	Gene ID	Chromosome location	CDS length	Numbers of AA	MW (kD)	pI
*CmDA1*	*MELO3C010913*	chr03:27671171:27676630:-	1443	480	55.41	5.78
*CmDA1-3*	*MELO3C007499*	chr08:3127292:3135702:-	1281	426	47.84	5.9
*CmDA1-2*	*MELO3C023896*	chr10:6975685:6980206:+	1416	471	53.27	6.84
*CmDA1-1*	*MELO3C020887*	chr11:3225210:3232204:+	1293	430	50.09	4.53

### 3.2 Phylogenetic and synteny analyses of the *DA1* gene family

To elucidate the phylogenetic relationships within the *DA1* family, a multiple sequence alignment was performed using ClustalW with protein sequences from *Cucumis melo* (melon), *Cucumis sativus* (cucumber), *Citrullus lanatus* (watermelon), *Arabidopsis thaliana*, and *Oryza sativa* (rice). A subsequent phylogenetic tree was constructed using the Neighbor-Joining method in MEGA 11 with 1,000 bootstrap replicates. Genome-wide identification revealed four DA1 genes in melon, five in cucumber, and five in watermelon.

**Figure 2:**
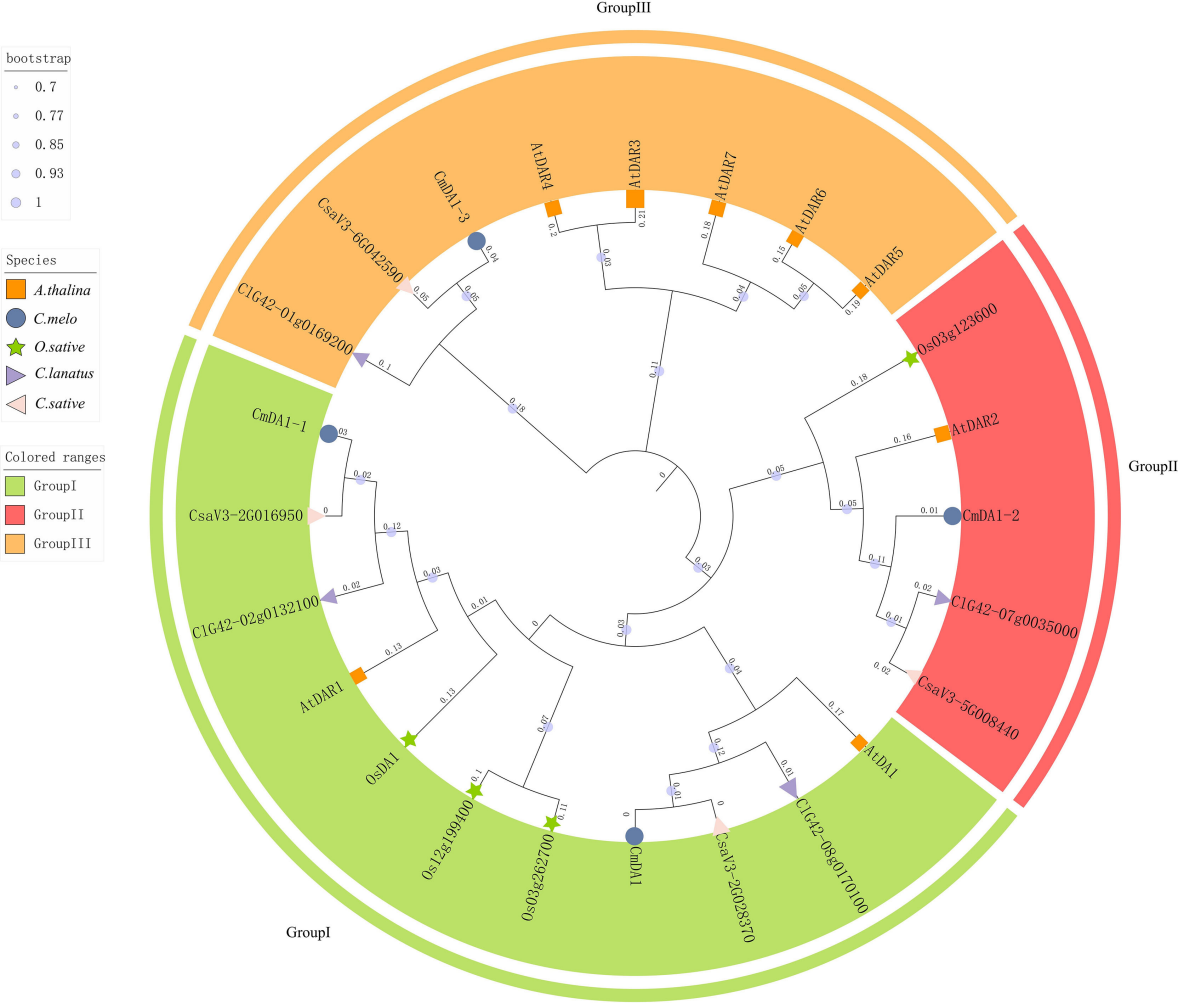
Phylogenetic tree of DA1 proteins from melon, cucumber, watermelon, *Arabidopsis*, and rice. The tree was constructed using the neighbor-joining method with 1,000 bootstrap replicates. The DA1 proteins are categorized into three distinct groups (I, II, III), each highlighted with a specific background color. Different species are marked with unique symbols: circles for melon (CmDA1), pink triangles for cucumber (CsDA1), purple triangles for watermelon (ClDA1), squares for *Arabidopsis* (AtDA1), and stars for rice (OsDA1).

The resulting phylogenetic tree classified all DA1 proteins into three distinct subfamilies (I–III) (**Figure 2**). Subfamily I comprised eleven members, including two from melon (CmDA1 and CmDA1-1), two from cucumber (CsaV3_2G028370 and CsaV3_2G016950), two from watermelon (ClG42_02g0132100 and ClG42_08g0170100.1), two from *Arabidopsis* (AtDA1 and AtDAR1), and three from rice (OsDA1, Os12g0596800, and Os03g0626600). Notably, CmDA1 clustered most closely with the cucumber gene CsaV3_2G028370, while CmDA1–1 formed a closest clade with CsaV3_2G016950. Subfamily II contained five members, featuring CmDA1–2 from melon, which showed close relationships with CsaV3_5G008440 from cucumber and ClG42_07g0035000 from watermelon. Subfamily III included eight members, consisting of five from *Arabidopsis* and one each from melon (CmDA1-3), cucumber (CsaV3_6G042590), and watermelon. Within this subfamily, CmDA1–3 shared the closest phylogenetic relationship with the cucumber ortholog CsaV3_6G042590.

**Figure 3:**
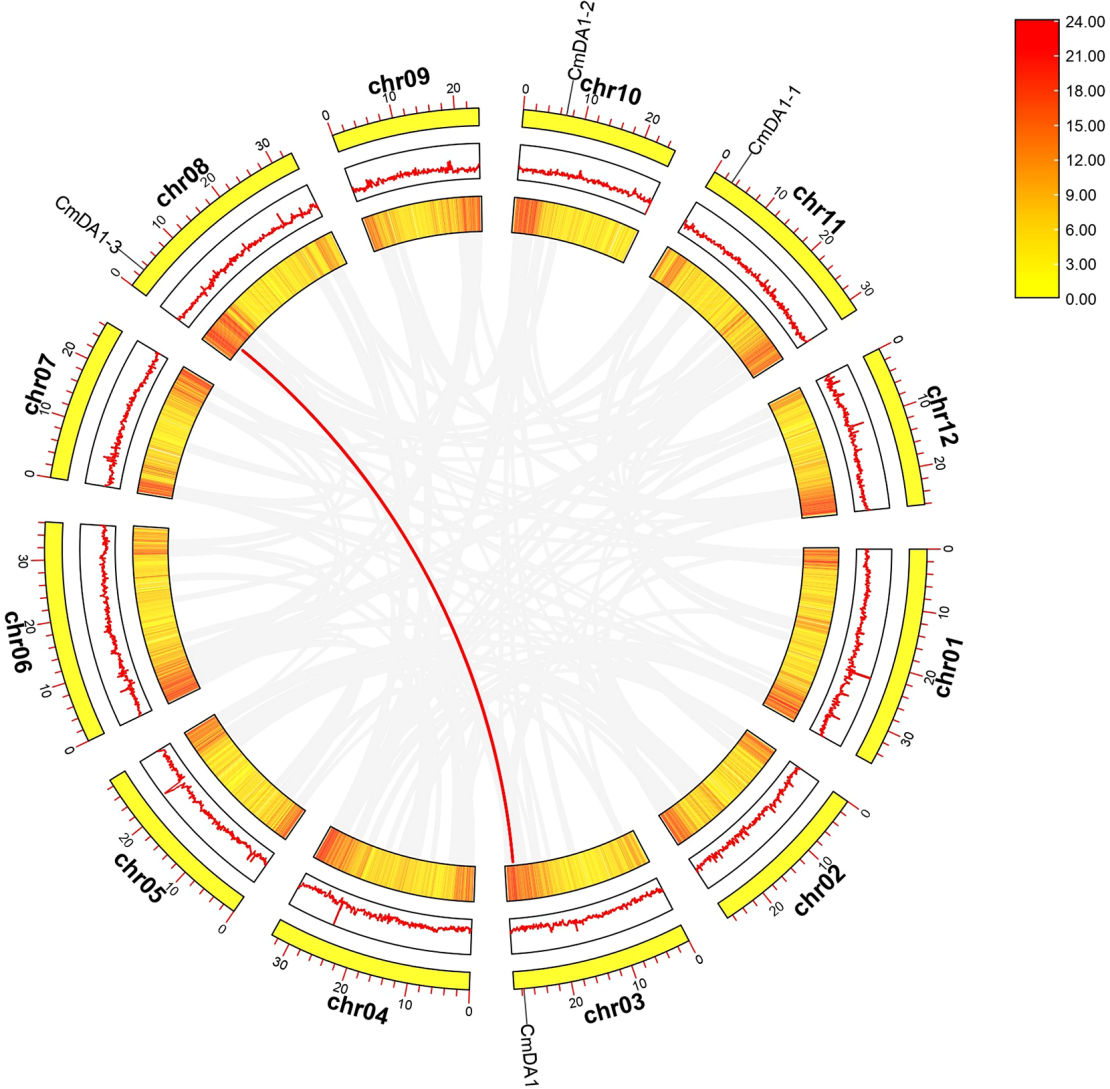
Synteny analysis of the *CmDA1* gene family in the melon genome. The circos plot displays the genomic distribution and duplication events of *CmDA1* genes. From the outermost to the innermost circle: the first circle represents the chromosomes of melon; the second circle shows the gene density distribution depicted as a red line plot; the third circle visualizes the gene density as a heatmap. The gray lines in the background represent all syntenic blocks in the melon genome, while the red line highlights the duplicated *CmDA1* gene pair.

**Figure 4:**
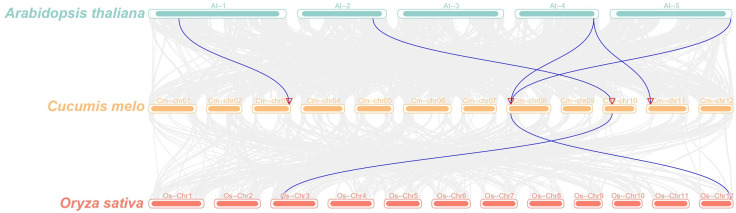
Comparative synteny analysis of *DA1* genes across melon, *Arabidopsis*, and rice. Gray lines in the background depict all systemic blocks between the genomes, while blue lines specifically highlight conserved systemic blocks containing homologous *DA1* gene pairs. Chromosome numbers for each species are labeled.

To investigate the evolutionary relationships of *DA1* genes across species, we performed collinearity analyses among *Arabidopsis*, *Cucumis melo*, and *Oryza sativa* (**Figure 3**). Interspecies analysis revealed five syntenic gene pairs between melon and *Arabidopsis* and two syntenic pairs between melon and *O. sativa*, suggesting potential functional importance of these conserved genes during melon evolution. Furthermore, intraspecies analysis within the melon genome identified only one syntenic relationship between *CmDA1* and *CmDA1-3* (**Figure 4**), indicating limited gene duplication events in the evolutionary history of the *DA1* family in melon.

### 3.3 Analysis of protein motifs and gene structure of *CmDA1* family members

**Figure 5:**
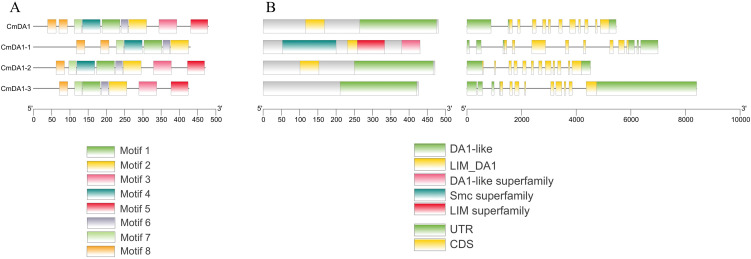
Conserved motifs and gene structure of *CmDA1* genes. **(A)** Distribution of conserved protein motifs. **(B)** Gene structure, showing the arrangement of exons and introns.

To elucidate the structural characteristics of the CmDA1 proteins, we analyzed their conserved motifs using the MEME suite (**Figure 5A**). Five motifs (1, 2, 6, 7, and 8) were consistently present across all four members, suggesting their fundamental role in DA1 protein function. Distinct distribution patterns were observed for other motifs: motifs 3 and 5 were exclusively shared by *CmDA1*, *CmDA1-2*, and *CmDA1-3*, while motif 4 was uniquely conserved in *CmDA1*, *CmDA1-1*, and *CmDA1-2*. These conserved yet differentiated motif patterns imply both functional conservation and potential specialization among *CmDA1* members.

Gene structure analysis revealed substantial diversity in exon-intron organization (**Figure 5B**). All *CmDA1* genes possess non-coding UTR regions and exhibit considerable variation in exon numbers, ranging from 8 (*CmDA1-1*) to 12 (*CmDA1-2*), with *CmDA1* and *CmDA1–3* containing 11 and 9 exons, respectively. Correspondingly, intron numbers varied from 7 to 11. The observed structural divergence in gene architecture suggests the possibility of functional differentiation within the *CmDA1* family.

### 3.4 Analysis of *cis*-acting elements in the promoters of *CmDA1* genes

**Figure 6:**
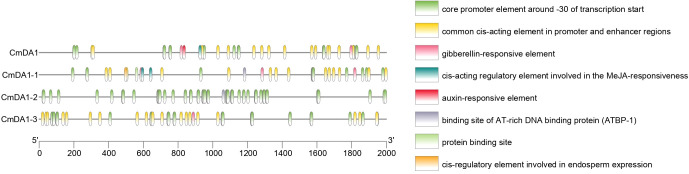
Distribution of predicted *cis*-acting elements in the promoter regions of *CmDA1* genes. The 2.0-kb genomic sequences upstream of the transcription start sites were analyzed. Identified elements are visualized as colored boxes and grouped by their predicted functions.

Analysis of the 2.0 kb promoter regions upstream of the *CmDA1* genes identified various *cis*-acting regulatory elements (**Figure 6**). All promoters contained core promoter elements around the -30 region and multiple hormone-responsive elements, including gibberellin-, auxin-, and MeJA-responsive motifs. The presence of these elements suggests potential regulation of *CmDA1* genes by phytohormones known to influence cell division and expansion. Additional motifs such as AT-rich DNA binding protein sites and endosperm expression-related elements were also detected. These findings indicate that *CmDA1* genes may be regulated by a complex network involving hormonal and developmental signals.

### 3.5 Tissue-specific and fruit developmental expression patterns of *CmDA1* genes

**Figure 7:**
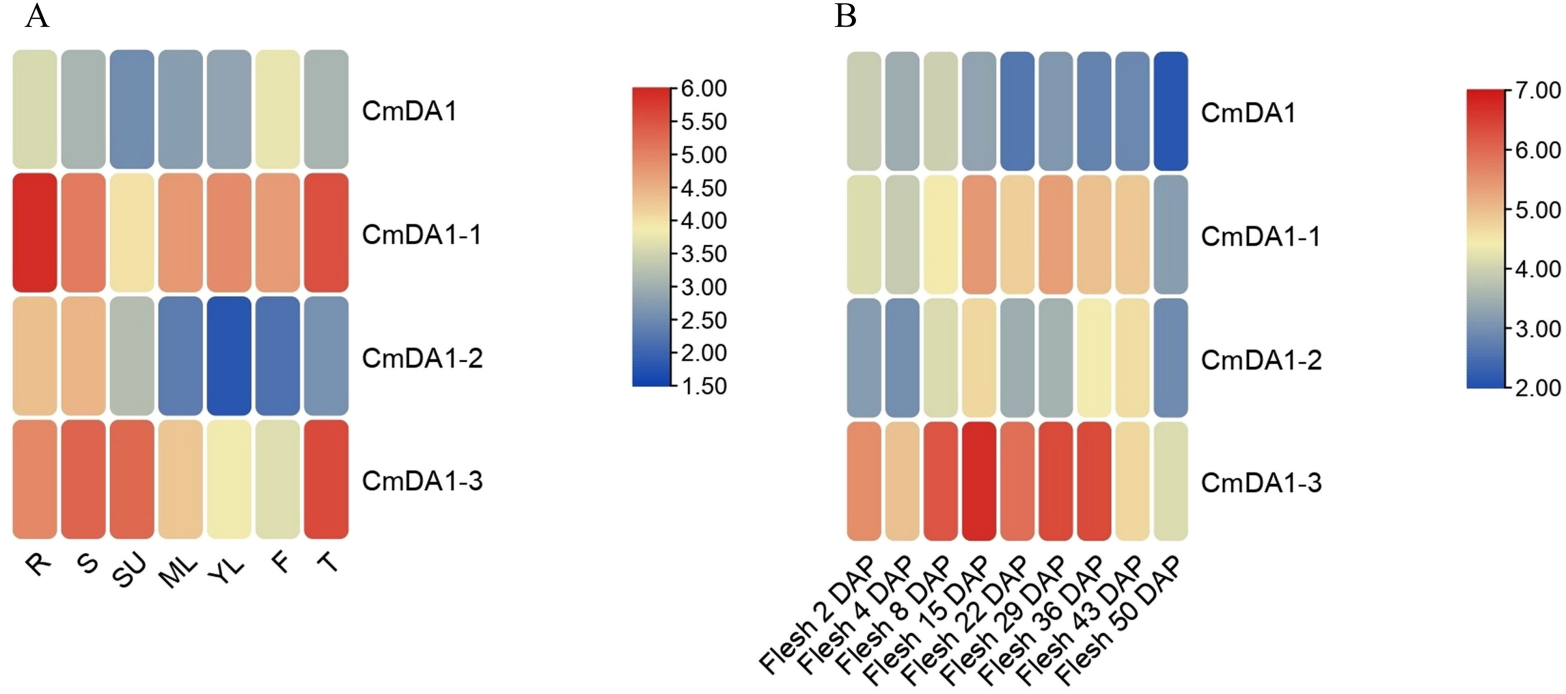
Expression patterns of *CmDA1* genes in melon. **(A)** Tissue-specific expression profiles across eight tissues: roots (R), stems (S), upper stems (SU), young leaves (YL), mature leaves (ML), flowers (F), fruits (Fr), and tendrils (T). **(B)** Expression dynamics during fruit flesh development at nine stages from 2 to 50 days after pollination (DAP). Expression levels are shown as log2(FPKM) values, with red indicating high expression and blue indicating low expression in the heatmaps.

To explore the spatial and temporal expression profiles of the *CmDA1* genes, we analyzed transcriptome data from eight distinct tissues and nine stages of fruit development. The expression patterns uncovered both conserved and divergent regulatory strategies among the four *CmDA1* genes (**Figure 7A**). In the tissue-specific analysis, each gene displayed unique expression preferences. *CmDA1* was predominantly expressed in roots and flowers, implying potential functions in root development and reproductive processes. *CmDA1–1* exhibited broad expression, with high transcript levels in seven of the eight tissues examined, except for upper stems. *CmDA1–2* showed preferential expression in roots and stems, indicating a possible role in vegetative growth regulation. Notably, *CmDA1–3* was highly expressed in six of the eight tissues, excluding young and mature leaves, suggesting it may act as a central regulator across multiple organs.

During fruit flesh development from 2 to 50 days after pollination (DAP), the four genes displayed distinct temporal expression dynamics (**Figure 7B**). *CmDA1* transcript levels gradually declined throughout fruit development, pointing to a potential early-stage role. *CmDA1–1* maintained consistently high expression from 15 to 43 DAP, with relatively lower expression at both earlier (2–8 DAP) and later (50 DAP) stages, indicating a mid-to-late phase-specific regulatory pattern. *CmDA1–2* exhibited peak expression at 15 and 43 DAP, with lower expression at other time points, indicative of stage-specific regulation. In contrast, *CmDA1–3* maintained consistently high expression during fruit development, with a marked upward trend from 2 to 36 DAP, underscoring its potential as a key persistent regulator of fruit maturation. Collectively, the distinct spatiotemporal expression profiles suggest functional divergence among the *CmDA1* members, and the broad tissue distribution combined with sustained expression during fruit development identifies *CmDA1–3* as a promising candidate for subsequent functional studies.

### 3.6 Quantitative expression analysis of *CmDA1* family members

**Figure 8:**
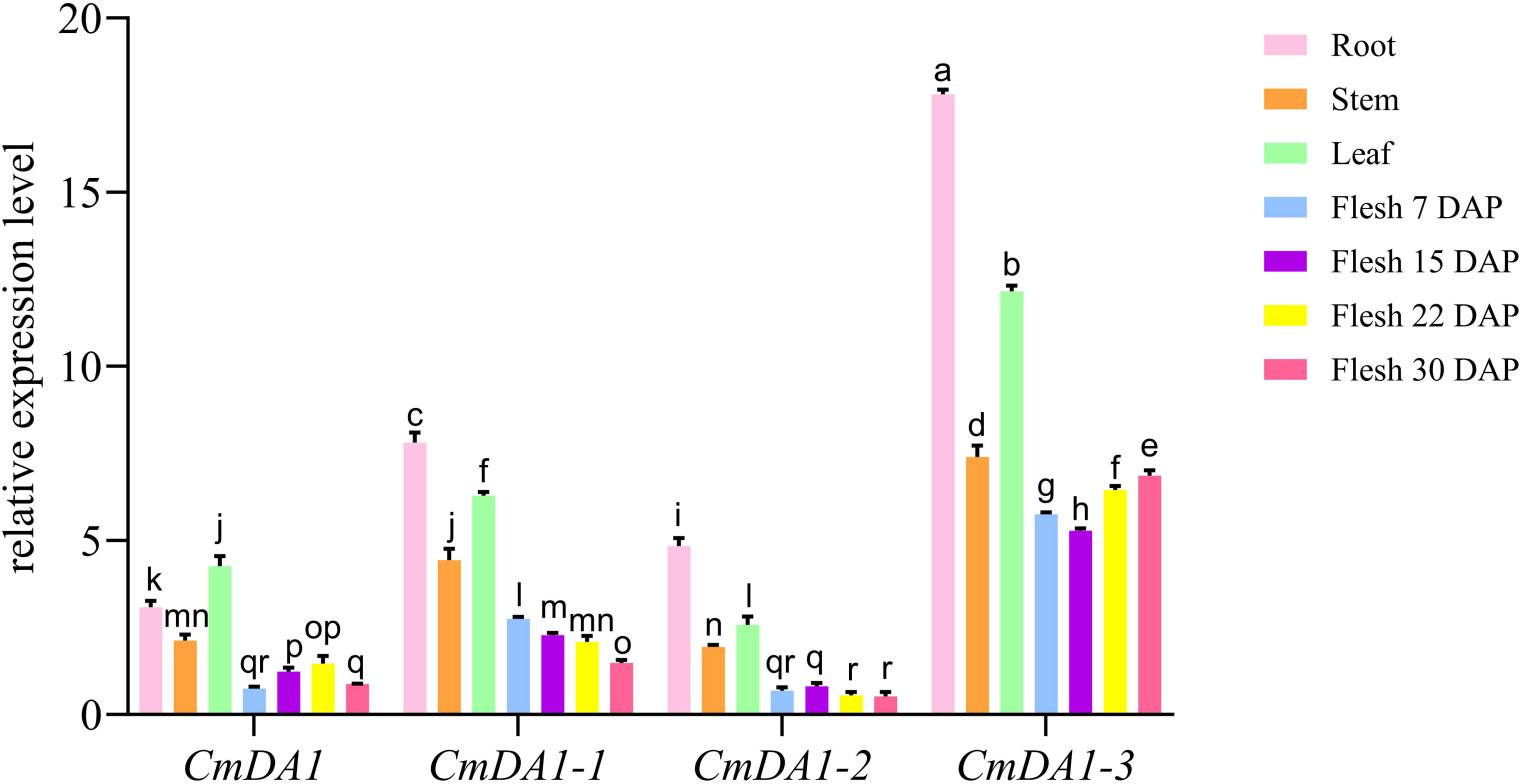
qRT–PCR analysis of *CmDA1* gene expression in HN15 melon. Expression levels of the four *CmDA1* genes across different tissues and fruit developmental stages (DAP) are presented as mean ± SEM (n=3). Lowercase letters indicate significant differences (*p* < 0.05, Duncan’s test) between samples.

To validate the expression patterns of *CmDA1* genes, we performed quantitative real-time PCR analysis in the melon inbred line HN15 across various tissues and fruit developmental stages (**Figure 8**). The results revealed distinct expression profiles among the four genes, with *CmDA1–3* consistently showing the highest expression levels across all examined samples.

In roots, all genes reached their maximum expression, with *CmDA1–3* showing particularly strong accumulation (mean: 17.81). *CmDA1–1* and *CmDA1–2* displayed moderate root expression (means: 7.81 and 4.83), while *CmDA1* showed the weakest expression (mean: 3.08). This pattern continued in stems and leaves, where *CmDA1–3* maintained the highest levels (means: 7.39 and 12.16), followed by *CmDA1-1* (means: 4.44 and 6.29).

During fruit development from 7 to 30 DAP, the genes displayed characteristic temporal patterns. *CmDA1–3* maintained stable, high expression throughout this period (means: 5.75 at 7 DAP to 6.86 at 30 DAP). In comparison, *CmDA1–1* showed moderate but gradually decreasing expression during fruit maturation (means: 2.75 at 7 DAP to 1.48 at 30 DAP). Both *CmDA1* and *CmDA1–2* maintained relatively low expression levels across all developmental stages, with *CmDA1–2* reaching particularly low levels at later stages (mean: 0.55 at 22 DAP). These qRT-PCR results corroborate the expression trends observed in our transcriptome data and highlight *CmDA1–3* as the most prominently expressed member in both vegetative tissues and developing fruits, suggesting its potential importance in melon growth and development.

## 4 Discussion

The *DA1* gene family encodes key negative regulators that final organ size by limiting cell proliferation duration in plants (Xu et al., 2024). Although this gene family has been characterized in several species, including *Arabidopsis* (Li et al., 2008; Zhao et al., 2014), rice (Shi et al., 2019; Li et al., 2024), maize (Xie et al., 2018), wheat (Liu et al., 2020), soybean (Zhao et al., 2015), rapeseed (Wang et al., 2017), cotton (Yang et al., 2021), and sweet potato (Zhou et al., 2024), its role in cucurbit crops, particularly in melon (*Cucumis melo*), remains largely unknown. Here, we present the first genome-wide analysis of the *DA1* family in melon, identifying four CmDA1 genes (**Figure 1**, **Table 1**). Our integrated analysis reveals both evolutionary conservation and functional diversification within this family, with *CmDA1–3* emerging as the most promising candidate for regulating fruit size in melon.

Phylogenetic analysis revealed that the CmDA1 proteins cluster into three distinct subfamilies with their orthologs from *Arabidopsis*, rice, cucumber, and watermelon (**Figure 2**), a classification consistent with previous reports in other species such as sweet potato (Zhou et al., 2024). The strong bootstrap-supported clustering of CmDA1 with AtDA1 and CmDA1–1 with AtDAR1 suggests functional conservation, possibly in the core complex where AtDA1 and AtDAR1 physically interact to synergistically restrict organ growth (Xia et al., 2013). Notably, CmDA1-3, which resides in subfamily III, forms a closest clade specifically with its ortholog from cucumber (CsaV3_6G042590) rather than with any *Arabidopsis* or rice member (**Figure 2**). This Cucurbitaceae-specific cluster, combined with the limited gene duplication in melon (**Figure 4**), indicates that while the gene lineage is conserved within cucurbits, *CmDA1–3* may have evolved specialized functions in melon. Similar functional diversification of DA1 members has been observed in other crops, such as cotton and sweet potato.

The observed phylogenetic relationships are further reflected in the gene structures and promoter compositions. While all CmDA1 proteins share a core set of conserved motifs, likely encompassing the critical UIM and LIM domains (**Figure 5A**), the presence of subfamily-specific motifs implies potential functional specialization. Most strikingly, we found substantial diversity in exon-intron architecture, with exon numbers ranging from 8 to 12 (**Figure 5B**). This structural divergence is a common evolutionary mechanism that can lead to functional differentiation within gene families, and has also been observed in the DA1 families of sweet potato and wheat (Liu et al., 2019; Zhou et al., 2024). Furthermore, promoter analysis revealed that all *CmDA1* genes contain multiple hormone-responsive elements, including those for gibberellin (GA), auxin, and jasmonic acid (JA) (**Figure 6**). These phytohormones are master regulators of cell division and expansion, indicating that the *CmDA1* genes may act as integrators of hormonal signals to fine-tune growth. Future research will use gene editing to validate these hormone-responsive elements and measure hormone levels during fruit development, ultimately elucidating how specific hormonal signals regulate the function of the CmDA1 gene family. The advancement of precision gene-editing tools now allows for the targeted manipulation of such cis-regulatory sequences, offering a direct path to decipher their *in vivo* functions (Qin et al., 2025). Additionally, the presence of MYB transcription factor binding sites suggests that *CmDA1* genes may be regulated by MYB proteins, which are known to participate in plant development and stress responses (Wang et al., 2021; Wu et al., 2024).This complex transcriptional regulation potential provides a molecular basis for the diverse expression patterns observed in our study.

Expression analysis confirmed the functional divergence suggested by phylogenetic and structural evidence. The four *CmDA1* genes showed unique expression patterns in different tissues and developmental stages (**Figures 7**, **8**). For example, *CmDA1* was mainly expressed in roots and flowers. This suggests it may function in reproductive development. In contrast, *CmDA1–2* was primarily expressed in roots and stems, indicating a role in vegetative growth. Such tissue-specific expression has also been observed in *DA1* genes from sweet potato and cotton (Yang et al., 2021; Zhou et al., 2024). Among all members, *CmDA1–3* displayed the most notable expression pattern, showing broad and high transcript abundance across vegetative tissues and maintaining strong expression throughout fruit development from 2 to 50 DAP (**Figures 7**, **8**). This persistent expression during fruit expansion and maturation is particularly striking. The *DA1* gene family has been reported to regulate organ and seed size in multiple species (Liu et al., 2019; Yang et al., 2021; Li et al., 2024). The sustained high expression of *CmDA1–3* thus suggests a potentially divergent function in melon. We propose that *CmDA1–3* may function as a modulator that fine-tunes the activity of other growth regulators, thereby promoting the extensive cell expansion required for melon fruit development. Together with its distinct phylogenetic position, the expression profile of *CmDA1–3* strongly indicates a specialized role in regulating fruit size in melon.

In conclusion, our study provides a comprehensive foundation for understanding the *DA1* gene family in melon. The combination of phylogenetic distinctness, complex regulatory potential, and dominant expression profile establishes *CmDA1–3* as a key candidate gene for fruit size regulation. Future research should focus on functional validation using CRISPR-Cas9-mediated mutagenesis, along with exploring natural allelic variation in this gene, to clarify its role and facilitate its use in melon breeding programs.

## Data Availability

The original contributions presented in the study are included in the article/**Supplementary Material**. Further inquiries can be directed to the corresponding authors.

## References

[B1] Bailey T. L. Boden M. Buske F. A. Frith M. Grant C. E. Clementi L. . (2009). MEME SUITE: tools for motif discovery and searching. Nucleic Acids Res. 37, W202–W208. doi: 10.1093/nar/gkp335, PMID: 19458158 PMC2703892

[B2] Dong H. Dumenil J. Lu F. H. Na L. Vanhaeren H. Naumann C. . (2017). Ubiquitylation activates a peptidase that promotes cleavage and destabilization of its activating E3 ligases and diverse growth regulatory proteins to limit cell proliferation in Arabidopsis. Genes Dev. 31, 197–208. doi: 10.1101/gad.292235.116, PMID: 28167503 PMC5322733

[B3] Du L. Li N. Chen L. L. Xu Y. X. Li Y. Zhang Y. Y. . (2014). The ubiquitin receptor DA1 regulates seed and organ size by modulating the stability of the ubiquitin-specific protease UBP15/SOD2 in arabidopsis. Plant Cell 26, 665–677. doi: 10.1105/tpc.114.122663, PMID: 24585836 PMC3967032

[B4] Gong P. Demuynck K. De Block J. Aesaert S. Coussens G. Pauwels L. . (2022). Modulation of the DA1 pathway in maize shows that translatability of information from Arabidopsis to crops is complex. Plant Sci. 321, 1–10. doi: 10.1016/j.plantsci.2022.111295, PMID: 35696903

[B5] Keren I. Lacroix B. Kohrman A. Citovsky V. . (2020). Histone deubiquitinase OTU1 epigenetically regulates DA1 and DA2, which control arabidopsis seed and organ size. Iscience 23, 1–13. doi: 10.1016/j.isci.2020.100948, PMID: 32169818 PMC7068640

[B6] Kesh H. Kaushik P. (2021). Advances in melon (Cucumis melo L.) breeding: An update. Scientia Hortic. 282, 1–10. doi: 10.1016/j.scienta.2021.110045

[B7] Krzywinski M. Schein J. Birol I. Connors J. Gascoyne R. Horsman D. . (2009). Circos: An information aesthetic for comparative genomics. Genome Res. 19, 1639–1645. doi: 10.1101/gr.092759.109, PMID: 19541911 PMC2752132

[B8] Kumar S. Stecher G. Tamura K. . (2016). MEGA7: molecular evolutionary genetics analysis version 7.0 for bigger datasets. Mol. Biol. Evol. 33, 1870–1874. doi: 10.1093/molbev/msw054, PMID: 27004904 PMC8210823

[B9] Letunic I. Bork P. (2021). Interactive Tree Of Life (iTOL) v5: an online tool for phylogenetic tree display and annotation. Nucleic Acids Res. 49, W293–W296. doi: 10.1093/nar/gkab301, PMID: 33885785 PMC8265157

[B12] Li Y. Zheng L. Corke F. Smith C. Bevan M. W. . (2008). Control of final seed and organ size by the DA1 gene family in Arabidopsis thaliana. Genes Dev. 22, 1331–1336. doi: 10.1101/gad.463608, PMID: 18483219 PMC2377187

[B10] Li C. Liu J. Zhang L. Y. Li T. Li H. Y. Liu B. . (2024). OsDA1 positively regulates grain width in rice. Crop J. 12, 92–101. doi: 10.1016/j.cj.2023.10.012

[B11] Li N. Li Y. (2014). Ubiquitin-mediated control of seed size in plants. Front. Plant Sci. 5. doi: 10.3389/fpls.2014.00332, PMID: 25071811 PMC4093792

[B13] Liu H. Li H. Hao C. Wang K. Wang Y. Qin L. . (2019). TaDA1, a conserved negative regulator of kernel size, has an additive effect with TaGW2 in common wheat (Triticum aestivum L.). Plant Biotechnol. J. 18, 1330–1342. doi: 10.1111/pbi.13298, PMID: 31733093 PMC7152612

[B14] Liu H. Li H. F. Hao C. Y. Wang K. Wang Y. M. Qin L. . (2020). TaDA1, a conserved negative regulator of kernel size, has an additive effect with TaGW2 in common wheat (Triticum aestivum L.). Plant Biotechnol. J. 18, 1330–1342. doi: 10.1111/pbi.13298, PMID: 31733093 PMC7152612

[B15] Marchler-Bauer A. Derbyshire M. K. Gonzales N. R. Lu S. N. Chitsaz F. Geer L. Y. . (2015). CDD: NCBI's conserved domain database. Nucleic Acids Res. 43, D222–D226. doi: 10.1093/nar/gku1221, PMID: 25414356 PMC4383992

[B16] Mayobre C. Pereira L. Eltahiri A. Bar E. Lewinsohn E. Garcia-Mas J. . (2021). Genetic dissection of aroma biosynthesis in melon and its relationship with climacteric ripening. Food Chem. 353, 129484. doi: 10.1016/j.foodchem.2021.129484, PMID: 33812162

[B17] Potter S. C. Luciani A. Eddy S. R. Park Y. Lopez R. Finn R. D. . (2018). HMMER web server: 2018 update. Nucleic Acids Res. 46, W200–W204. doi: 10.1093/nar/gky448, PMID: 29905871 PMC6030962

[B18] Qin J. Wang P. Ma W. Zhang C.-J. . (2025). Genome editing in polyploid crops: progress, challenges, and prospects. Genomics Commun. 2, 0–0. doi: 10.48130/gcomm-0025-0022

[B19] Shahwar D. Khan Z. Park Y. . (2023). Molecular marker-assisted mapping, candidate gene identification, and breeding in melon (Cucumis melo L.): A review. Int. J. Mol. Sci. 24, 1–22. doi: 10.3390/ijms242015490, PMID: 37895169 PMC10607903

[B20] Shi C. L. Ren Y. L. Liu L. L. Wang F. Zhang H. Tian P. . (2019). Ubiquitin specific protease 15 has an important role in regulating grain width and size in rice. Plant Physiol. 180, 381–391. doi: 10.1104/pp.19.00065, PMID: 30796160 PMC6501108

[B22] Vanhaeren H. Nam Y. J. De Milde L. Chae E. Storme V. Weigel D. . (2017). Forever young: the role of ubiquitin receptor DA1 and E3 ligase BIG BROTHER in controlling leaf growth and development. Plant Physiol. 173, 1269–1282. doi: 10.1104/pp.16.01410, PMID: 28003326 PMC5291030

[B21] Vanhaeren H. Chen Y. Vermeersch M. De Milde L. De Vleeschhauwer V. Natran A. . (2020). UBP12 and UBP13 negatively regulate the activity of the ubiquitin-dependent peptidases DA1, DAR1 and DAR2. Elife 9, e52276. doi: 10.7554/eLife.52276, PMID: 32209225 PMC7141810

[B25] Wang Y. Tang H. DeBarry J. D. Tan X. Li J. Wang X. . (2012). MCScanX: a toolkit for detection and evolutionary analysis of gene synteny and collinearity. Nucleic Acids Res. 40, e49–e49. doi: 10.1093/nar/gkr1293, PMID: 22217600 PMC3326336

[B23] Wang J. L. Tang M. Q. Chen S. Zheng X. F. Mo H. X. Li S. J. . (2017). Down-regulation of BnDA1, whose gene locus is associated with the seeds weight, improves the seeds weight and organ size in Brassica napus. Plant Biotechnol. J. 15, 1024–1033. doi: 10.1111/pbi.12696, PMID: 28097785 PMC5506660

[B24] Wang X. P. Niu Y. L. Zheng Y. . (2021). Multiple functions of MYB transcription factors in abiotic stress responses. Int. J. Mol. Sci. 22, 1–14. doi: 10.3390/ijms22116125, PMID: 34200125 PMC8201141

[B26] Wu X. Y. Xia M. Su P. Zhang Y. F. Tu L. C. Zhao H. . (2024). MYB transcription factors in plants: A comprehensive review of their discovery, structure, classification, functional diversity and regulatory mechanism. Int. J. Biol. Macromolecules 282, 1–22. doi: 10.1016/j.ijbiomac.2024.136652, PMID: 39427786

[B27] Xia T. Li N. Dumenil J. Li J. Kamenski A. Bevan M. W. . (2013). The ubiquitin receptor DA1 interacts with the E3 ubiquitin ligase DA2 to regulate seed and organ size in arabidopsis. Plant Cell 25, 3347–3359. doi: 10.1105/tpc.113.115063, PMID: 24045020 PMC3809536

[B28] Xie G. N. Li Z. X. Ran Q. J. Wang H. Zhang J. R. . (2018). Over-expression of mutated ZmDA1 or ZmDAR1 gene improves maize kernel yield by enhancing starch synthesis. Plant Biotechnol. J. 16, 234–244. doi: 10.1111/pbi.12763, PMID: 28557341 PMC5785342

[B30] Xu L. He Y. Tang L. Xu Y. Zhao G. . (2022). Genetics, genomics, and breeding in melon. Agronomy 12, 2891–2903. doi: 10.3390/agronomy12112891

[B29] Xu F. Dong H. X. Guo W. J. Le L. Jing Y. X. Fletcher J. C. . (2024). The trxG protein ULT1 regulates Arabidopsis organ size by interacting with TCP14/15 to antagonize the LIM peptidase DA1 for H3K4me3 on target genes. Plant Commun. 5, 1–20. doi: 10.1016/j.xplc.2024.100819, PMID: 38217289 PMC11009162

[B31] Yang S. Huang L. Song J. Liu L. Bian Y. Jia B. . (2021). Genome-wide analysis of DA1-like genes in gossypium and functional characterization of GhDA1-1A controlling seed size. Front. Plant Sci. 12. doi: 10.3389/fpls.2021.647091, PMID: 34093610 PMC8173226

[B32] Yano R. Nonaka S. Ezura H. . (2018). Melonet-DB, a Grand RNA-Seq gene expression atlas in melon (Cucumis melo L.). Plant Cell Physiol. 59, e4–e4. doi: 10.1093/pcp/pcx193, PMID: 29216378

[B33] Ye J. McGinnis S. Madden T. L. . (2006). BLAST: improvements for better sequence analysis. Nucleic Acids Res. 34, W6–W9. doi: 10.1093/nar/gkl164, PMID: 16845079 PMC1538791

[B35] Zhao M. He L. L. Gu Y. Z. Wang Y. Chen Q. S. He C. Y. . (2014). Genome-wide analyses of a plant-specific LIM-domain gene family implicate its evolutionary role in plant diversification. Genome Biol. Evol. 6, 1000–1012. doi: 10.1093/gbe/evu076, PMID: 24723730 PMC4007552

[B34] Zhao M. Gu Y. Z. He L. L. Chen Q. S. He C. Y. . (2015). Sequence and expression variations suggest an adaptive role for the DA1-like gene family in the evolution of soybeans. BMC Plant Biol. 15, 120. doi: 10.1186/s12870-015-0519-0, PMID: 25975199 PMC4432951

[B36] Zhou Z. L. Huang J. Z. Wang Y. H. He S. X. Yang J. Wang Y. . (2024). Genome-wide identification and expression analysis of the DA1 gene family in sweet potato and its two diploid relatives. Int. J. Mol. Sci. 25, 1666–1681. doi: 10.3390/ijms25053000, PMID: 38474246 PMC10931741

